# Erv1 of *Arabidopsis thaliana* can directly oxidize mitochondrial intermembrane space proteins in the absence of redox-active Mia40

**DOI:** 10.1186/s12915-017-0445-8

**Published:** 2017-11-08

**Authors:** Valentina Peleh, Flavien Zannini, Sandra Backes, Nicolas Rouhier, Johannes M. Herrmann

**Affiliations:** 1 Cell Biology, University of Kaiserslautern, Erwin-Schrödinger-Strasse 13, 67663 Kaiserslautern, Germany; 2 Unité Mixte de Recherches 1136 Interactions Arbres-Microorganismes, Université de Lorraine/INRA, Faculté des sciences et technologies, 54500 Vandoeuvre-lès-Nancy, Nancy, France

**Keywords:** Disulfide bond formation, Eukaryotic evolution, Intermembrane space, Mitochondria, Oxidative protein folding, Protein translocation

## Abstract

**Background:**

Many proteins of the mitochondrial intermembrane space (IMS) contain structural disulfide bonds formed by the mitochondrial disulfide relay. In fungi and animals, the sulfhydryl oxidase Erv1 ‘generates’ disulfide bonds that are passed on to the oxidoreductase Mia40, which oxidizes substrate proteins. A different structural organization of plant Erv1 proteins compared to that of animal and fungal orthologs was proposed to explain its inability to complement the corresponding yeast mutant.

**Results:**

Herein, we have revisited the biochemical and functional properties of *Arabidopsis thaliana* Erv1 by both in vitro reconstituted activity assays and complementation of *erv1* and *mia40* yeast mutants. These mutants were viable, however, they showed severe defects in the biogenesis of IMS proteins. The plant Erv1 was unable to oxidize yeast Mia40 and rather even blocked its activity. Nevertheless, it was able to mediate the import and folding of mitochondrial proteins.

**Conclusions:**

We observed that plant Erv1, unlike its homologs in fungi and animals, can promote protein import and oxidative protein folding in the IMS independently of the oxidoreductase Mia40. In accordance to the absence of Mia40 in many protists, our study suggests that the mitochondrial disulfide relay evolved in a stepwise reaction from an Erv1-only system to which Mia40 was added in order to improve substrate specificity.

**Graphical Abstract:**
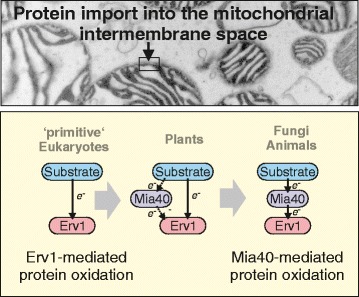
The mitochondrial disulfide relay evolved in a step-wise manner from an Erv1-only system.

**Electronic supplementary material:**

The online version of this article (doi:10.1186/s12915-017-0445-8) contains supplementary material, which is available to authorized users.

## Background

Two compartments of the eukaryotic cell, namely the endoplasmic reticulum (ER) and the mitochondrial intermembrane space (IMS), contain disulfide relays to introduce structural disulfide bonds into proteins and to facilitate oxidative protein folding [1]. In the ER, proteins are oxidized by direct interaction with members of the protein disulfide isomerase (PDI) family, which are maintained in an oxidized state by the sulfhydryl oxidase Ero1 [2]. Thus, PDIs provide substrate specificity whereas Ero1 initially ‘generates’ the disulfides [3, 4].

The function of the mitochondrial disulfide relay is less well understood. The sulfhydryl oxidase of the IMS, Erv1, is a flavoprotein just like Ero1. Although not structurally related, the architecture of the flavodomain of Erv1 family members is similar to that of Ero1, presumably as a result of convergent evolution [5–8]. Unlike PDIs, the mitochondrial oxidoreductase Mia40 has no thioredoxin fold and its structure is entirely different to that of PDI. Mia40 has a hydrophobic substrate-binding cleft that recognizes patterns of hydrophobic residues in helical regions of its substrates, referred to as mitochondrial intermembrane space sorting signal (MISS) or intermembrane space targeting signal (ITS) sequences [9–17]. This interaction drives the translocation of IMS proteins across the outer membrane [14, 18, 19]. During or directly following the import reaction, Mia40 forms mixed covalent dimers with its substrates, which can be stable for seconds to minutes [12, 13, 20–25]. This lasting interaction is very different to the very rapid disulfide exchange reaction of PDI with its substrates [26].

Reduced Mia40 is re-oxidized by Erv1 in a reaction that mimics the Mia40-substrate interaction [9]. The Erv1-catalyzed oxidation of Mia40 is highly efficient, such that in vivo Mia40 is predominantly or exclusively present in the oxidized state [20, 25, 27–30]. Reconstitution experiments proved that Mia40 and Erv1 are the only two proteins required to drive efficient oxidation of IMS proteins in vitro [21, 28, 31].

Mia40 is conserved among animals, fungi, and plants but absent in many ‘more primitive’ eukaryotes such as in trypanosomes [32] or in kinetoplastids [33]. Presumably, Mia40 was never present in these groups but it cannot be excluded that certain organisms initially contained Mia40 but secondarily lost it during evolution.

In contrast, genes for Erv1 homologs were identified ubiquitously in genomes of mitochondria-containing eukaryotes [34]. However, the structural organization of Erv1 proteins considerably differs among organisms of different eukaryotic phyla. Studies in *Arabidopsis thaliana* suggested that, in plants, Mia40 (*At*Mia40) is located both in mitochondria and peroxisomes (due to a C-terminal SKL targeting signal) and is dispensable for IMS import [35]. Still, the *A. thaliana* Erv1 (*At*Erv1) was found to be essential and critical for mitochondrial biogenesis.

Since a detailed functional analysis of Erv1 can hardly be performed in plants, we decided to express *At*Erv1 in *erv1* and *mia40* yeast mutants lacking a functional disulfide relay in the IMS and to re-examine if and why it could not complement these mutants [35, 36]. While we observed that *At*Erv1 did not cooperate with yeast Mia40 but rather blocked its function, it still mediated protein import into the IMS, unexpectedly interacting directly with imported IMS proteins and facilitating their oxidative folding. Thus, upon expression of *At*Erv1, the redox-active CPC motif on the yeast Mia40 became dispensable as the plant Erv1 can fold some client proteins directly. Overall, this suggests that, during evolution, Mia40 was added to an Erv1-only system at a later stage, presumably in order to improve substrate specificity and isomerization of more complex substrates.

## Results

### Erv1 proteins of plants, animals, and fungi differ in their domain organization

**Fig. 1:**
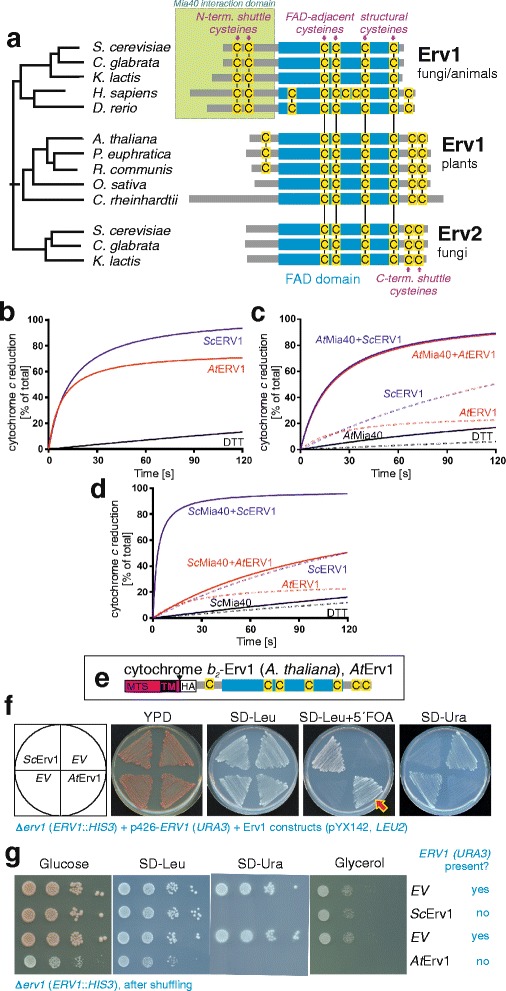
The yeast *ERV1* gene can be deleted upon expression of its *Arabidopsis* homolog. **a** Phylogeny and structural comparison of members of the Erv1 family (see Additional file 1: Figure S1 for details). An overview of the protein sequences is shown, in which all cysteine residues are indicated in yellow. **b** Reduction of cytochrome *c* (40 μM) by purified yeast or *A. thaliana* Erv1 (8 μM) in the presence of 100 μM DTT. A control measurement without Erv1 is shown (DTT). **c** Reduction of cytochrome *c* (40 μM) by 50 μM DTT alone or in the presence of 20 μM *At*Mia40, 8 μM *At*Erv1, 8 μM yeast Erv1 (*Sc*Erv1), or 20 μM *At*Mia40 combined with 8 μM *At*Erv1 or 8 μM *Sc*Erv1. **d** Reduction of cytochrome *c* (40 μM) by 50 μM DTT alone, or in the presence of 20 μM *Sc*Mia40, 8 μM *At*Erv1, 8 μM *Sc*Erv1, or 20 μM *Sc*Mia40 combined with 8 μM *At*Erv1 or 8 μM *Sc*Erv1. **e** Schematic representation of the *At*Erv1 protein used in this study. *MTS* mitochondrial targeting signal, *TM* transmembrane domain of cytochrome *b*_*2*_ (residues 1–169) to verify IMS targeting, *HA* hemagglutinin tag. **f** By use of a plasmid shuffle strategy, a *URA3* plasmid for the expression of yeast Erv1 could be replaced by a *LEU2* plasmid harboring a gene for the synthesis of *At*Erv1 (red arrow). *EV* empty vector. **g** Strain in which the *URA3* plasmid was replaced by a plasmid expressing yeast Erv1 or AtErv1, grown to log phase. Ten-fold serial dilutions were dropped on the indicated media. Whereas cells expressing the yeast Erv1 were able to respire, the *At*Erv1 mutant did not grow on non-fermentative carbon sources such as glycerol

The Erv1 protein family is characterized by a conserved domain that mediates the electron transfer between a bound FAD cofactor and a surface-exposed CxxC motif (Fig. 1a, Additional file 1: Figure S1). This domain is present in the Erv1 proteins of mitochondria, in the Erv2 proteins present in the ER of fungi, and in members of the rather diverse QSOX group [37, 38]. However, the regions that flank this conserved FAD domain in these proteins differ considerably. In the Erv1 proteins of fungi and animals, an N-terminal flexible region serves as an essential interaction arm that shuttles electrons between Mia40 and the FAD domain of Erv1 [9, 21]. Erv1 proteins of plants and Erv2 proteins lack this region but contain a C-terminal redox-active disulfide that, at least in the case of Erv2, serves as an electron shuttle [36, 39].

The ability of Erv1 to shuttle electrons from electron donors such as DTT or Mia40 to its electron acceptor cytochrome *c* can be measured by following cytochrome *c* reduction at 550 nm. We purified recombinant yeast Erv1 and *At*Erv1 produced in *E. coli* and monitored the reduction of cytochrome *c* over time in the presence of 100 μM DTT (Fig. 1b). Both proteins efficiently accelerated the rate of cytochrome *c* reduction, although the yeast Erv1 showed a somewhat higher efficiency in this reaction. Then, *At*Mia40 was introduced in these assays after reducing the DTT concentration to 50 μM to limit its direct reaction with Erv1 while maintaining the Mia40 catalytic disulfide reduced (Fig. 1c). Using *At*Mia40, yeast Erv1 and *At*Erv1 could transfer electrons to cytochrome *c* with comparable efficiency. However, when yeast Mia40 was used, only yeast Erv1 was able to catalyze cytochrome *c* reduction (Fig. 1d), suggesting that *At*Erv1 cannot efficiently accept electrons from yeast Mia40. Overall, the in vitro measurements indicated that the Erv1 proteins from yeast and *A. thaliana* exhibited comparable in vitro capacity to reduce cytochrome *c* with the notable difference that *At*Erv1 does not accept electrons from yeast Mia40.

In order to test whether, despite its different domain organization, plant Erv1 can functionally replace the well-characterized Erv1 protein of yeast, we generated a *LEU2* plasmid for the expression of *At*Erv1 fused to the IMS-targeting sequence of cytochrome *b*_2_ for adequate targeting followed by a hemagglutinin tag present in the N-terminal region for detection (Fig. 1e). This differs from previous studies that did not verify the correct localization of the proteins and used either an intact sequence or added a shorter cytochrome *b*_2_ pre-sequence and fused *At*Erv1 with or without a C-terminal His-tag [36]. We transformed this plasmid into a Δ*erv1* mutant that contained the yeast *ERV1* gene on a *URA3* plasmid. Through growth on 5′ fluoroorotic acid, we counter-selected against the *URA3* plasmid, yielding viable cells. Viable cells were also obtained with the yeast *ERV1* gene on a *LEU2* plasmid but not with the empty plasmid (Fig. 1f). We concluded that *At*Erv1 can replace the Erv1 protein of yeast. However, this strain was unable to grow on glycerol, indicating that it was unable to respire (Fig. 1g). Thus, obviously, although *At*Erv1 can take over an essential function of Erv1, it is not able to replace the yeast protein in its role in the biogenesis of the respiratory chain.

### Mitochondria expressing *At*Erv1 show severely reduced levels of Mia40 substrates

**Fig. 2:**
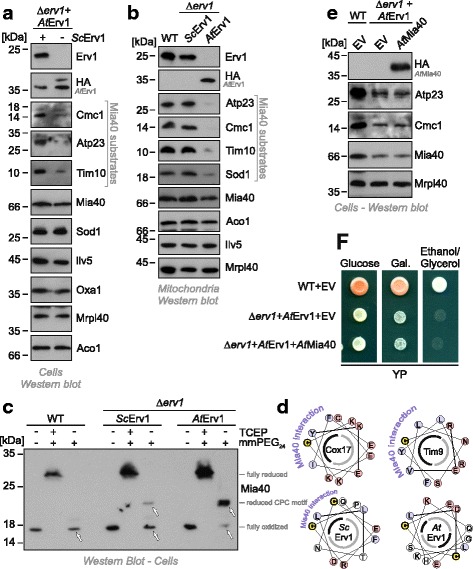
*At*Erv1 fails to oxidize the catalytic cysteines in the CPC motif of Mia40. Western blot analysis of extracts of whole cells (**a**) or isolated mitochondria (**b**) of the indicated strains show a strong depletion of Mia40 substrates in strains that express *At*Erv1 in the absence of yeast Erv1. Panel a shows the shuffle strain before and after shuffling out the *Sc*Erv1-encoding URA3 plasmid. **c** To monitor the redox state of Mia40 in the different strains, proteins of the indicated strains were TCA-precipitated (in order to ‘freeze’ the redox state of thiol groups), denatured in SDS, treated with the reducing agent tris(2-carboxyethyl)phosphine (TCEP) and the alkylating compound methyl-polyethylene glycol_24_-maleimide (mmPEG_24_), and visualized by SDS-PAGE and western blotting. For this experiment a Mia40 variant was used that lacked the long membrane linker, which leads to much more reliable results in this kind of shift assay [18, 25]. TCEP reduced all thiols in Mia40 such that its six cysteines were alkylated, resulting in a shift of approximately 12 kDa (2 kDa per mmPEG_24_). In wild-type cells, in the absence of TCEP, Mia40 was not shifted since all cysteines were oxidized (arrowhead). Additionally, in the Δ*erv1* mutant that was complemented by yeast Erv1, the cysteine residues of Mia40 remained largely inaccessible. In the *At*Erv1-expressing mutant, however, almost no oxidized Mia40 was detectable. The shift by 4 kDa corresponds to the alkylation of the two redox-active cysteines of Mia40, indicative of the reduced form of Mia40. It should be noted that the two structural disulfides that are critical for the formation of the substrate-binding domain of Mia40 were formed in this mutant. **d** Helical wheel representation of the Mia40 interaction region in Cox17, Tim9, yeast Erv1 (*Sc*), and *At*Erv1 [9, 10, 14, 17]. The hydrophobic (black) and hydrophilic (grey) faces of the helix are indicated as half circles. Note that the docking cysteines in Cox17 and Tim9, as well as cysteines of the shuttle disulfide in yeast Erv1 (yellow), are part of an amphipathic helix structure whereas cysteines of the shuttle disulfide of *At*Erv1 are not. **e**, **f** Levels of IMS proteins were analyzed by western blotting (**e**). Growth of the indicated mutants on non-fermentable medium (**f**). *Gal* galactose

Next, we assessed whether *At*Erv1 can replace the yeast Erv1 protein in its function in mitochondrial protein import. To this end, we analyzed the levels of different mitochondrial proteins in whole cells (Fig. 2a) or isolated mitochondria (Fig. 2b) of the different *At*Erv1-expressing mutants. The levels of matrix-targeted proteins (Ilv5, Oxa1, Mrpl40, or Aco1) were similar in these samples. However, in mutants expressing *At*Erv1 but no yeast Erv1, the levels of Mia40 substrates, such as Atp23, Tim10 or Cmc1, were strongly reduced. In these cells, Sod1 levels were normal in whole cell extracts whereas the protein was almost absent from mitochondria, which confirms that the biogenesis of the IMS-located fraction of Sod1 requires the disulfide relay whereas the cytosolic Sod1 does not [40–42]. Additionally, Mia40 levels were reduced in the *At*Erv1 mitochondria, highlighting problems in its oxidative folding that may cause an Yme1-mediated instability (Fig. 2b).

Moreover, we expressed *At*Erv1 in a temperature-sensitive *erv1* mutant (*erv1-ts*) [43] and isolated mitochondria from cells that were shifted to restrictive conditions for 16 h. Again, the levels of the Mia40 substrates Atp23 and Cmc1 were severely reduced, whereas low amounts of the essential IMS protein Tim10 were still detected (Additional file 2: Figure S2A). Thus, the *At*Erv1 protein can facilitate the import of low levels of small Tim proteins and therefore exhibits the essential function of Erv1. However, *At*Erv1-expressing mitochondria lack Cmc1, an IMS protein required for the assembly of cytochrome oxidase [44–46]. Accordingly, we observed considerably reduced levels of subunit 2 of cytochrome oxidase (Cox2, Additional file 2: Figure S2B), although this mitochondrially encoded protein was synthesized at normal levels (Additional file 2: Figure S2C). Thus, the absence of IMS-located biogenesis factors of cytochrome oxidase explains the inability of the *At*Erv1 mutant to respire.

### *At*Erv1 does not oxidize yeast Mia40 in vivo

The severe defects observed in the *At*Erv1-expressing mutants and the in vitro activity results prompted us to test whether *At*Erv1 can oxidize the yeast Mia40 protein in vivo. To this end, we analyzed the redox state of Mia40 in the different mutants by an alkylation shift assay based on the modification of reduced but not oxidized thiols by methyl-polyethylene glycol_24_-maleimide (mmPEG_24_, Fig. 2c). In wild-type cells, Mia40 is almost exclusively present in the oxidized state [21, 47] and its six cysteine residues can only be alkylated after reducing its three disulfide bonds with tris(2-carboxyethyl)phosphine, a thiol-free chemical reductant (Fig. 2c, wild-type). In contrast, the two catalytic cysteine residues of Mia40 remained accessible in the *At*Erv1-expressing mutant and almost no oxidized Mia40 was observed. Thus, *At*Erv1 is extremely inefficient in oxidizing the yeast Mia40 protein, which may be explained by the fact that the shuttle disulfide of *At*Erv1 is not part of an amphipathic helix structure (Fig. 2d) that could serve as a Mia40 interaction region.

The inability of *At*Erv1 to cooperate with yeast Mia40 could point to an incompatibility of the fungal and plant systems. However, neither the decreased levels of Mia40 substrates (Fig. 2e) nor the growth defects on glycerol (Fig. 2f) of the *At*Erv1-expressing mutant were suppressed by co-expressing *At*Mia40. Apparently, the *At*Erv1 protein does not productively cooperate with Mia40.

This is further supported by the observation that the *At*Erv1-expressing mutant was hypersensitive to DTT (Additional file 3: Figure S3A, B), which counteracts disulfide bond formation by the mitochondrial disulfide relay [21]. Re-oxidation of Erv1 can occur either in a cytochrome *c*-independent reaction in which oxygen is directly reduced to hydrogen peroxide or in a cytochrome *c*-mediated reaction that can also occur under anaerobic conditions [48–50]. Since the *At*Erv1-expressing mutant grows efficiently also in the absence of oxygen (Additional file 3: Figure S3C), we regard it as unlikely that the defect in this strain is due to an incompatibility of *At*Erv1 with yeast cytochrome *c*, but rather due to an incompatibility of *At*Erv1 with Mia40.

### Mitochondria of the *At*Erv1-expressing mutant show defects in the import of IMS proteins

**Fig. 3:**
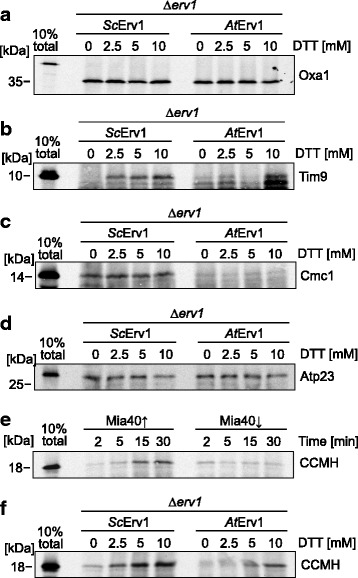
Mitochondria expressing *At*Erv1 fail to import the Mia40 substrates Tim9, Cmc1, and CCMH. **a**–**f** The indicated proteins were synthesized in the presence of [^35^S]-methionine in reticulocyte lysate and incubated with mitochondria isolated from the indicated strains for the times indicated. Non-imported material was removed by treatment with proteinase K in all samples shown in this figure. Mitochondria were washed, re-isolated and subjected to SDS-PAGE and autoradiography; 10% of the radiolabeled protein used per time point was loaded as a control

Next, we directly tested the ability of mitochondria from Δ*erv1* cells that expressed either yeast Erv1 or *At*Erv1 to import proteins in vitro. To this end, we purified mitochondria and incubated them with radiolabeled precursor proteins destined for the matrix (Oxa1, Fig. 3a) or the IMS (Tim9, Cmc1, Atp23, Fig. 3b–d) in the presence of different concentrations of DTT. Non-imported material was removed by protease treatment before samples were analyzed by SDS-PAGE and autoradiography. Oxa1 was efficiently imported into both mitochondria, verifying that they were import-competent. However, the import of Tim9 and Cmc1 was almost completely blocked. This confirms previous studies showing that the redox state of Mia40 strongly influences the import efficiency, even though the oxidation of the catalytic disulfide bond in Mia40 is not essential for the import of Tim9, but only for its subsequent folding and assembly [14, 18, 19, 51]. In contrast to Tim9 and Cmc1, the Mia40 substrate Atp23 was efficiently imported into the *At*Erv1-expressing mitochondria. Atp23 differs from Tim9 and Cmc1 by the fact that its cysteine residues are not required for its import since a cysteine-free mutant of Atp23 was shown to be still efficiently imported in a strictly Mia40-dependent manner [19, 51, 52]. However, without the structural disulfide bonds, Atp23 is unstable and rapidly degraded by Yme1.

The proteome of plant mitochondria, and presumably also that of their IMS, differs considerably from that of fungi and animals [53, 54]. For example, bacteria, plant mitochondria, and chloroplasts use common systems for the biogenesis of *c*-type cytochromes, which differs considerably from the *c*-type cytochrome biogenesis machinery of animals and fungi [55]. The plant system, but not that of animals and fungi, contains the protein CCMH, which contributes to the heme incorporation. CCMH has a conserved protein domain exposed into the IMS and contains two cysteines in a CxxC motif [56]. We wondered whether the yeast system could be used to test whether CCMH is a substrate of the mitochondrial disulfide relay. To this end, we incubated radiolabeled *Arabidopsis* CCMH with isolated mitochondria of Mia40-containing and Mia40-depleted mitochondria. We observed that the import of CCMH was strongly reduced in mitochondria in which Mia40 was depleted (Fig. 3e), suggesting that this protein is a substrate of the mitochondrial disulfide relay. Further, this plant substrate was imported much more efficiently in mitochondria expressing the yeast Erv1 than in those expressing *At*Erv1 (Fig. 3f). Thus, the poor performance of the *At*Erv1-expressing mutant is not caused by an incompatibility of yeast substrates with *At*Erv1 but rather by an incompatibility of *At*Erv1 with Mia40.

### *At*Erv1 binds efficiently to yeast Mia40 but has a dominant-negative activity in yeast mitochondria

**Fig. 4:**
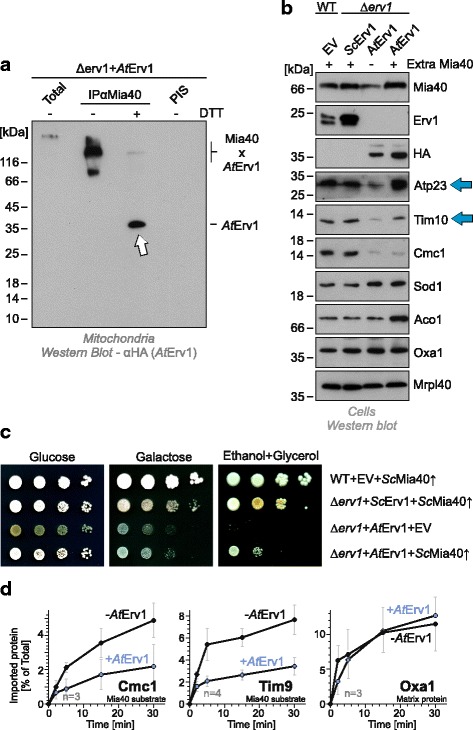
*At*Erv1 exhibits a dominant-negative activity on yeast Mia40. **a** Mitochondria isolated from a Δ*erv1* strain expressing *At*Erv1 were treated with 1 mM cleavable cross-linker dithiobis succinimidyl propionate for 15 min at 25 °C and lysed with 1% SDS. The extract was used for immunoprecipitation with Mia40-specific antibodies or with pre-immune serum (PIS). Hemagglutinin-tagged *At*Erv1 protein was visualized by western blotting. Arrows indicate immunoprecipitated *At*Erv1. Total samples contain 10% of the material used per immunoprecipitation reaction. **b** A multicopy plasmid carrying the yeast *MIA40* gene was transformed into wild-type or Δ*erv1* cells. Whole cell extracts were prepared and analyzed by western blotting. Note that the levels of Atp23 and Tim10 were largely restored upon overexpression of Mia40 despite the absence of yeast Erv1 in these mutants (blue arrows). **c** An extra copy of yeast Mia40 (*Sc*Mia40) partially rescues the growth defect of the *At*Erv1 mutant on non-fermentative medium. **d** Mitochondria were isolated from wild-type cells lacking or carrying an expression plasmid for *At*Erv1. The Mia40 substrates Cmc1 and Tim9, as well as the matrix protein Oxa1, were incubated with these mitochondria at 25 °C for the times indicated. Non-imported material was removed by protease treatment. The amounts of imported radiolabeled proteins were quantified. Mean values and standard deviations of at least three replicates are shown

The inability of *At*Erv1 to oxidize yeast Mia40 might be due either to an inability of both proteins to interact or to an unproductive interaction. Co-immunoprecipitation experiments with Mia40-specific antibodies efficiently pulled down a fraction of *At*Erv1 after stabilization of the interaction with the cleavable crosslinker dithiobis succinimidyl propionate (Fig. 4a). As expected, newly imported radiolabeled Mia40 was efficiently recovered in a complex with *At*Erv1 (Additional file 4: Figure S4A). From this we concluded that *At*Erv1 and yeast Mia40 are able to interact and it is apparently not the lack of binding that prevented *At*Erv1 from functionally replacing yeast Erv1, but *At*Erv1 rather appears to block Mia40 activity.

In order to mitigate a potentially dominant-negative activity of *At*Erv1 on Mia40, we transformed the mutants with a plasmid harboring an extra copy of Mia40 (Fig. 4b). Surprisingly, overexpression of yeast Mia40 indeed partially suppressed the negative effect of *At*Erv1 as it restored the levels of some IMS proteins (Tim10 and Atp23, Fig. 4b) and allowed slow growth of the strains on non-fermentable carbon sources (Fig. 4c).

Furthermore, the dominant-negative effect of *At*Erv1 was very obvious when the protein was expressed in wild-type mitochondria containing a functional yeast Erv1 protein. The presence of *At*Erv1 strongly impaired the import of Mia40 substrates such as Cmc1 or Tim9, but not that of the matrix protein Oxa1 (Fig. 4d). In summary, we concluded that *At*Erv1 did interact with Mia40 but rather blocked its activity, presumably by competitive inhibition of its hydrophobic substrate binding domain.

### *At*Erv1 can oxidize IMS proteins in the absence of a redox-active Mia40

**Fig. 5:**
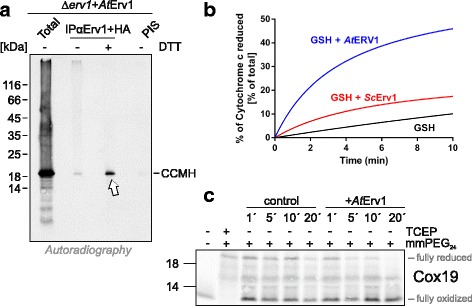
Plant Erv1 can directly oxidize IMS proteins. **a** Radiolabeled *A. thaliana* CCMH was incubated for 2 min with isolated mitochondria from the *At*Erv1-expressing Δ*erv1*. Mitochondria were treated with 400 μM cleavable cross-linker dithiobis succinimidyl propionate for 5 min at 25 °C and lysed with 1% SDS. The extract was used for immunoprecipitation with a combination of Erv1- and hemagglutinin-specific antibodies or with pre-immune serum (PIS). The crosslinker was cleaved with DTT when indicated. The radiolabeled protein was visualized by autoradiography. Total samples contain 10% of the material used per immunoprecipitation reaction. Arrow depicts radiolabeled CCMH pulled down with *At*Erv1. **b** Cytochrome *c* reduction by plant and yeast Erv1 with glutathione (GSH) as an electron donor. The reduction of cytochrome *c* (40 μM) was followed at 550 nm over 10 min after incubation with 5 mM GSH in the presence or absence of 8 μM *At*Erv1 or *Sc*Erv1. All of the reduced cytochrome *c* was obtained with 50 μM DTT, as shown in Fig. 1c. **c** In vitro-translated radioactive Cox19 was incubated in the absence or presence of 30 μM purified *At*Erv1 for the indicated times. Subsequently, samples were TCA-precipitated, treated with 15 mM mmPEG_24_ for 1 h at 25 °C, and then subjected to non-reducing SDS-PAGE and analyzed by autoradiography

It is possible that *At*Erv1 may be able to rescue the Δ*erv1* mutant without productively interacting with Mia40 through the direct interaction of Erv1 with incoming polypeptides, bypassing the need for Mia40. To test this, we immunoprecipitated *At*Erv1 from mitochondria in which we had imported radiolabeled CCMH. Indeed, CCMH was efficiently pulled down in a DTT-sensitive manner (Fig. 5a), confirming the direct *At*Erv1-substrate interaction. In contrast, no CCMH was recovered with Mia40-specific antibodies, suggesting that, in the presence of *At*Erv1, the import of CCMH occurs independently of Mia40 (Additional file 5: Figure S5). Further, not only CCMH but also the yeast proteins Tim9 and Tim17 were found to interact with *At*Erv1, although the efficiency of the crosslinking was lower than that of the plant substrate (Additional file 4: Figure S4B, C). Additionally, small amounts of yeast Erv1 were found to be in contact with newly imported Tim9 and Tim17, in line with previous studies showing that small Tim proteins and Tim17 are oxidized in yeast mitochondria that lack redox-active Mia40 [18, 57].

In order to test whether *At*Erv1 is able to oxidize thiol-containing peptides, we employed the cytochrome *c* assay to monitor the oxidation of glutathione with *At*Erv1 and yeast Erv1 as a control (Fig. 5b). We observed that *At*Erv1 oxidized glutathione, resulting in cytochrome *c* reduction. In contrast, the yeast Erv1 protein was extremely inefficient in its interaction with glutathione, suggesting that *At*Erv1 can directly oxidize this small peptide whereas the yeast Erv1 protein does not or only to a minimal extent.

We then tested whether *At*Erv1 is able to oxidize a well-established Mia40 substrate. To this end, we incubated radiolabeled Cox19 in the absence or presence of purified *At*Erv1 (Fig. 5c). At different time points, proteins were precipitated by acid treatment and denatured, and reduced thiols were alkylated with mmPEG_24_. In the presence of *At*Erv1, but not in its absence, reduced Cox19 protein was efficiently depleted from the reaction, indicating that *At*Erv1 is indeed able to oxidize this yeast protein, at least in vitro. It should be noted that yeast Erv1 has been previously shown to slowly oxidize Cox19 in vitro [18, 21, 23, 58].

**Fig. 6:**
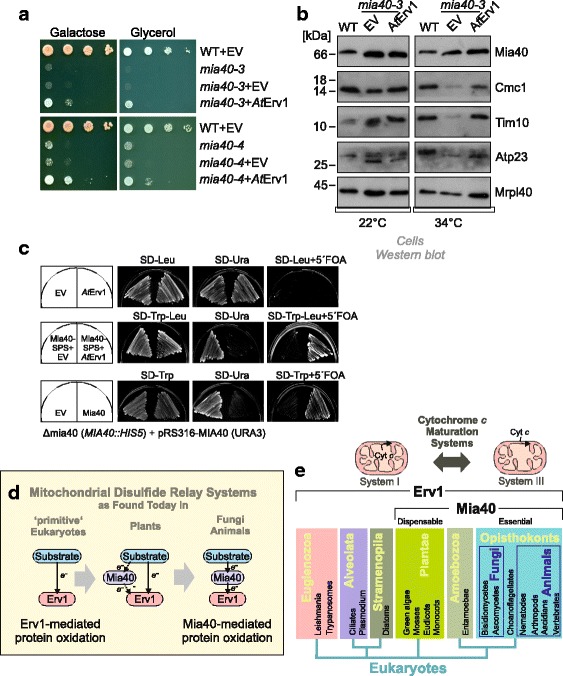
**a**, **b** The expression of *At*Erv1 in the temperature-sensitive Mia40 strains *mia40-3* and *mia40-4* allows cell growth at a restrictive temperature (**a**) and restores the level of Mia40 substrates in the IMS of mitochondria (**b**). **c** Δ*mia40* cells containing *MIA40* on an *URA3* plasmid were transformed with plasmids to express *At*Erv1 alone (upper row), *At*Erv1 in combination with Mia40-SPS (middle row), or wild-type Mia40 as a positive control (lower row). The ability of the strains to grow upon loss of *URA3* plasmid was tested on 5-fluoroorotic acid. Co-expression of *At*Erv1 and Mia40-SPS resulted in viable cells. **d**, **e** Many ‘primitive’ eukaryotic phyla (such as euglenozoa, alveolata, and stramenopila) lack Mia40 and protein oxidation should be carried out by Erv1 directly. In plants, a gene for Mia40 is present but not essential, as substrate oxidation can still occur in a Mia40-independent manner. In opisthokonts, both Erv1 and Mia40 are crucial for protein oxidative folding. We propose that the initial disulfide relay only employed an Erv1-like oxidase and was thus similar to the situation still found in some protists

Next, we used genetics to test whether *At*Erv1 was indeed able to bypass the need for Mia40. To this end, we expressed *At*Erv1 in the temperature-sensitive Mia40 mutants *mia40-3* and *mia40-4* [23]. *At*Erv1 partially suppressed the growth defect of these strains at restrictive growth conditions (Fig. 6a, Additional file 6: Figure S6A) and restored the levels of Mia40 substrates, such as Cmc1 and Tim10, to some degree (Fig. 6b, Additional file 6: Figure S6B).

Since it cannot be excluded that the Mia40 of the temperature-sensitive mia40 mutant still has some residual activity even at restrictive conditions, we decided to test whether a redox-active Mia40 can be completely removed in the presence of *At*Erv1 in the IMS. We therefore performed plasmid shuffling experiments in the absence or presence of a redox-inactive Mia40 mutant in which both cysteines of the CPC motif were replaced by serine residues (Mia40-SPS) [18]. Expression of *At*Erv1 allowed the loss of *MIA40* as long as Mia40-SPS was co-expressed (Fig. 5d). Thus, a combination of Mia40-SPS, which mediates the import but not the oxidation of proteins [18], and of *At*Erv1, which subsequently oxidizes imported proteins, is obviously sufficient to promote the biogenesis of IMS proteins (Additional file 6: Figure S6C). This demonstrates that *At*Erv1 can take over the essential function of the mitochondrial disulfide system in the IMS as long as the hydrophobic substrate-binding region of Mia40 facilitates protein translocation across the outer membrane.

## Discussion

*A. thaliana* mutants lacking Mia40 are viable and it was proposed that the mitochondrial disulfide relay system can function without Mia40 or that an additional, not yet characterized, oxidoreductase makes Mia40 dispensable [35]. The results presented in this study show that *At*Erv1 can oxidize substrate proteins directly in a Mia40-independent manner. In contrast, Erv1 proteins of fungi and animals were suggested to oxidize substrate proteins predominantly or exclusively via the oxidoreductase Mia40. However, recent studies suggest that also in yeast, Erv1 can oxidize specific substrates such as the inner membrane protein Tim17 [57] and, with low efficiency, typical Mia40 substrates, at least in the background of Mia40 mutants that lack the redox-active cysteine pair [18].

Although the molecular details of this *At*Erv1-mediated protein oxidation have to be better delineated, we report here that *At*Erv1 can directly bind to newly imported IMS proteins, thereby forming mixed disulfides. This promotes their import and oxidative folding in the IMS even in strains that lack a functional Mia40 protein. Thus, the mitochondrial disulfide relay of plants obviously differs from that of fungi and animals as it allows the direct electron flow from substrates to *At*Erv1 (Fig. 6d). Possibly, *At*Mia40 might still handle specific, non-essential substrates or carry out another function such as the isomerization of IMS proteins. Such a substrate-specific function could explain the decreased complex I activity observed in Mia40 plant mutant lines [35]. Moreover, a recent study showed that *At*Mia40 can form a complex with the mitochondrial phosphatase SLP2 and proposed a regulatory, again substrate-specific, role of *At*Mia40 in plant mitochondria [59].

Interestingly, many eukaryotic groups, including ciliates, diatoms, or parasites such as *Leishmania*, *Trypanosoma*, or *Plasmodium*, lack genes for Mia40 homologs but still contain Erv1 (Fig. 6e) [32–34, 60]. Thus, the mitochondrial disulfide relay obviously started from an Erv1-only system in which Erv1, similar to the QSOX proteins of the secretory pathway [38, 61], directly catalyzed protein oxidation (Fig. 5e). Mia40 was presumably added later, initially as a dispensable player. In fungi and animals, however, Mia40 became essential and Erv1 lost its ability to interact directly with IMS proteins. It should be noted that we do not propose that animals and fungi developed from plants; however, given the intermediate stage of the plant system compared to that of protists on the one side and of animals/fungi on the other, we assume that the disulfide relay in animals and fungi developed from a situation that was similar to that still operating in mitochondria of *Arabidopsis*.

Why was Mia40 added into the mitochondrial disulfide relay during eukaryotic evolution? Oxidoreductases can provide substrate specificity, thereby targeting the oxidative potential of sulfhydryl oxidases to specific clients. This is most obvious for the disulfide relay of the ER, where the sulfhydryl oxidase Ero1 presumably oxidizes only one single substrate, PDI, which oxidizes many different substrate proteins (often employing even additional PDI homologs) [3, 4]. The substrate specificity of Mia40 was well characterized and IMS proteins with disulfide bonds show MISS/ITS signals to ensure their specific recognition by Mia40 [14, 17]. It is unclear why an increased substrate specificity became necessary during evolution; however, the change in the *c*-type cytochrome biogenesis system coincided with the addition of Mia40 [55]. Cytochrome *c* is by far the most abundant protein of the IMS. It is characterized by two reduced cysteines in a CxxC motif to which heme is covalently attached during cytochrome biogenesis. Oxidation of these cysteines prevents cytochrome maturation, and bacteria employ a specific reduction system in order to keep apocytochrome *c* reduced. In mitochondria of protists and plants (as well as in bacteria), cytochrome *c* is secreted across the inner membrane into the IMS and matured by a complex machinery employing many conserved factors (called system-1 and system-2) [62]. Several of these components are mitochondrially encoded due to their hydrophobic nature. In contrast, in fungi and animals, which have a considerably smaller mitochondrial genome, apocytochrome *c* is imported from the cytosol and matured by a single enzyme, cytochrome *c* heme lyase. Perhaps, the increased substrate specificity of Mia40 made a much simpler maturation system for *c*-type cytochromes possible and allowed the further reduction of the set of mitochondrially encoded genes [62, 63].

The results of this study also nicely demonstrate that, for the mitochondrial disulfide relay, the proper interplay between its two components Mia40 and Erv1 is essential. Although the catalytic activity of *At*Erv1 in disulfide bond formation is comparable to that of yeast Erv1 when using DTT, *At*Erv1 was unable to productively interact with Mia40. *At*Erv1 did not efficiently oxidize yeast Mia40, which prevented the import of IMS proteins. The observation that overexpression of Mia40 mitigated the defects of the *At*Erv1-expressing mutant and that the expression of *At*Erv1 exhibited a dominant-negative activity despite yeast Erv1 being present suggests that *At*Erv1 arrested the Mia40 reaction cycle. *At*Erv1 binds efficiently to Mia40 but fails to oxidize it, presumably due to its different domain organization compared with yeast Erv1. Thus, the results shown in this study are compatible with a model according to which *At*Erv1 acts as a competitive inhibitor of Mia40, blocking its substrate-binding site without oxidizing its CPC motif.

It should be noted that the phenotype of the *At*Erv1-expressing mutant cannot be simply explained by the insufficient oxidation of Mia40 as a Mia40-SPS mutant (which lacks the catalytic disulfide) still imports proteins into the IMS, but fails to oxidize them [18]. As a consequence, these unfolded proteins fail to assemble and are rapidly degraded by the IMS-AAA protease Yme1 [19, 51, 52].

IMS proteins, such as Cox17 or Tim9, bind to Mia40 via hydrophobic interactions of a helical sequence known as MISS or ITS signal [14, 17]. These signals are characterized by an amphipathic helix, which places the attached cysteine residue within a hydrophobic patch (Fig. 2d). The yeast Erv1 protein has a similar, though less pronounced, sequence believed to mimic the MISS/ITS signal of Mia40 substrates (Fig. 2d). In contrast, *At*Erv1 lacks such a pattern. Here, the shuttle cysteines are separated by four residues such that a disulfide bond is not compatible with the formation of a helical structure, which should prevent *At*Erv1 to properly align its shuttle disulfide with the CPC motif of Mia40 and thus to oxidize yeast Mia40 (Fig. 2d).

In fungi and animals, Mia40 and Erv1 cooperate in the import and folding of proteins into the IMS. The use of a combination of a Mia40-SPS mutant, which traps import intermediates but is unable to oxidize them, and of the *At*Erv1 protein, which promotes substrate oxidation in a Mia40-independent reaction, showed that these two functions of the disulfide relay can be completely separated. This will provide an excellent system in order to dissect the individual reactions of the biogenesis of IMS proteins in mitochondria in greater mechanistic detail.

## Conclusions

Disulfide relays stabilize protein structures by oxidative protein folding. The present study shows that the mitochondrial disulfide relay of plants is much simpler than that of previously studied systems and may resemble the oxidation machinery of early eukaryotes; its sulfhydryl oxidase *At*Erv1 oxidizes substrates directly, making an oxidoreductase obsolete.

## Methods

### Yeast strains and plasmids

Yeast strains used in this study were based on the wild-type strains W303 and YPH499. Shuffle strains for *ERV1* and *MIA40*, as well as *erv1-ts*, *mia40-3*, and *mia40-4* mutants, have been previously described [18, 21, 23, 58]. Yeast strains were either grown in synthetic media containing 2% glucose or galactose, or in YP (1% yeast extract, 2% peptone) medium containing 2% galactose, glucose, glycerol, or ethanol [64].

For expression of the *At*Erv1 variant, the *At*Erv1 sequence was amplified from leaf Arabidopsis cDNA using the primers: forward 5' CCCCGGATCCTATCCTTACGACGTGCCTGACTACGCCGGTGAGAAGCCATGGCAGCCAC 3' and reverse 5' CCCCGTCGACTTAAAAGTCCATAGAAGTTCCATG 3', introducing an N-terminal hemagglutinin tag. The amplified fragment was cloned in frame into the *BamHI* and *SalI* restriction sites of the pYX142 vector (Addgene) containing the sequence coding for the amino acids 1–167 of cytochrome *b*_2_ (comprising its mitochondrial targeting sequence, the transmembrane domain and the heme-binding domain) into *EcoRI* and *BamHI* restriction sites.

To express *At*Mia40, the sequence corresponding to the protein sequence of residues 2–161 was cloned using *BamHI* and *BstX1* restriction sites in frame into the single copy vectors pRS314, pRS315, or pRS316 harboring *MIA40* promoter and a sequence corresponding to the amino acid residues 1–70 of yeast Mia40 [18]. To overexpress yeast Mia40, the entire *MIA40* gene, including the promoter and terminator, was cloned by PCR between *SacI* and *SalI* restriction sites into the multi-copy vectors pRS424 and pRS426.

For in vitro transcription/translation of CCMH, the sequence corresponding to amino acid residues 1–159 was amplified from an *A. thaliana* cDNA using the primers: forward 5′ CCCCCGAATTCGCCACCATGGAGAAAACAGACGAAGAG 3′ and reverse 5′ CCCCCGGATCCCTACCGGTTGAGCCATCTCC 3′ and cloned into the *EcoRI* and *BamHI* sites of a pGEM4 vector (Promega).

Experimental procedures on the isolation of mitochondria, the import of radiolabeled precursor proteins, immunoprecipation and western blotting have been reported previously [15].

### Cloning, expression, and purification of recombinant proteins in *E. coli*

The *At*Erv1 sequence (without the region coding for the first 70 amino acids) and the *At*Mia40 sequence (without the region coding for the first 55 amino acids) were amplified by PCR from *A. thaliana* leaf cDNA using the following pairs of primers: *At*Erv1 forward 5′ CCCCCCCCATATGACTGGTCCTGTGACTAAAGAG 3′ and *At*Erv1 reverse 5′ CCCCGGATCCCTAAAAGTCCATAGAAGT 3′ and *At*Mia40 forward 5′ CCCCCCCCATATGGAGTCTCTTGAAGCC 3′ and *At*Mia40 reverse 5′ CCCCGGATCCCTAAAGCTTGGAATTGCC 3′ and cloned in pET12a and pET15b plasmids, respectively (Novagen). The pET24a-*Sc*Erv1 and pGEX6-*Sc*Mia40 expression plasmids have been previously described [18, 21, 23, 58]. Protein production was achieved in the *Escherichia coli* BL21(DE3) strain containing pSBET plasmids using a culture protocol previously described [65]. *At*Erv1 was purified in three successive steps (ammonium sulfate precipitation, ACA44 gel filtration and DEAE-Sepharose), whereas *At*Mia40 and *Sc*Erv1 were purified in a single step on His-Select® Nickel affinity gel (Sigma-Aldrich) from the soluble part of the bacterial extract following procedures already described [65]. After dialysis against a 30 mM Tris-HCl (pH 8.0), 1 mM EDTA buffer, proteins were stored at −20 °C. The purification of the GST-*Sc*Mia40 fusion was performed on Glutathione Sepharose 4B (GE Healthcare, product code 17-0756-01) following the manufacturer’s recommendations. The GST-tag cleavage was performed in the a 50 mM Tris-HCl (pH 7.0), 150 mM NaCl, 1 mM EDTA, and 1 mM DTT buffer by adding the recommended amount of Precision Protease on the glutathione sepharose resin for overnight incubation at 4 °C. The cleaved protein was eluted using a 50 mM Tris-HCl (pH 8.0), 1 mM EDTA, and 150 mM NaCl buffer, dialyzed against a 30 mM Tris-HCl (pH 8.0) and 1 mM EDTA buffer, and finally stored at −20 °C in 50% glycerol. All protein concentrations were determined using the respective theoretical extinction coefficients at 280 nm calculated using the Expasy Protparam tool (http://web.expasy.org/protparam/).

Antibodies used in this study were generated in rabbits against recombinantly expressed and purified Erv1 or Mia40 [20, 21] or commercially obtained from Roche (Anti_HA-Peroxidase; Cat. No. 12013819001; Antibody ID AB_390917).

### Alkylation shift experiments for redox state detection

To analyze the redox state of cysteine residues in whole cells, mitochondrial proteins were precipitated with TCA, denatured with SDS, and incubated with 15 mM mmPEG_24_ (Thermo Scientific product # 22713) as described [15].

### Reduction of cytochrome *c*

The reduction of cytochrome *c* from equine heart (40 μM, SIGMA-Aldrich, product code C7752) was followed by recording changes in absorbance at 550 nm using a Cary 50 Variant-Agilent spectrophotometer. The reactions were performed in a 50 mM phosphate buffer at pH 7.4 and 1 mM EDTA, and started by adding DTT (50 or 100 μM) to the cuvettes containing various combinations of purified recombinant proteins (Erv1 (8 μM) and Mia40 (20 μM) from both *S. cerevisiae* and *A. thaliana*).

## Additional files

Additional file 1: Figure S1. Related to Fig. 1. Alignment of members of the Erv1 family. The sequences were compared using Clustal Omega with standard settings of the program. (DOCX 1001 kb)

Additional file 2: Figure S2. Related to Fig. 2. Mitochondria of the *At*Erv1-expressing *erv1-ts* mutant mutant show strongly reduced levels of Mia40 substrates and no functional cytochrome oxidase. **A** The temperature-sensitive *erv1*-*ts* mutant was transformed with plasmids to express yeast Erv1 or *At*Erv1. Cells were shifted to restrictive conditions at 34 °C for 16 h before mitochondria were isolated and analyzed by western blotting. **B** *At*Erv1-expressing cells show strongly reduced steady-state levels of Cox2, a central, mitochondrially encoded subunit of cytochrome oxidase. **C** Mitochondrial translation products were radiolabeled in cells after blocking cytosolic translation with cycloheximide. Cells were incubated for 15 min at 30 °C in the presence of [^35^S]-methionine. Radiolabeled proteins were separated by SDS-PAGE and visualized by autoradiography. (EPS 6782 kb)

Additional file 3: Figure S3. Related to Fig. 2. The *At*Erv1-expressing *Δerv1* mutant shows increased sensitivity to DTT. **A**, **B** For the experiment shown in panel A, 10 μL of 3 M DTT was added to the filter paper placed in the middle of the plates. For the experiment shown in panel B, the following solutions were used: water, 1 M diamide, 1 M DTT or 3 M DTT (10 µL each) or 30% H2O2 (5 μL) as indicated. Cells were grown at 30 °C for 3 days. **C** Wild-type and Δ*erv1* cells expressing yeast Erv1or *At*Erv1 were incubated on glucose plates in the presence (blue color of the indicator strip) or absence (white color of the indicator strip) of oxygen for 5 days at 30 °C. (EPS 142732 kb)

Additional file 4: Figure S4. Related to Fig. 4. *At*Erv1 directly interacts with Tim9 and Tim17. **A** Radiolabeled Mia40 was incubated for 2 min with isolated *erv1*-*ts* mitochondria containing or lacking hemagglutinin (HA)-tagged *At*Erv1. Mitochondria were re-isolated and lysed with 1% SDS. The extract was used for immunoprecipitation with HA-specific antibodies or with pre-immune serum (PIS). Disulfide bonds were reduced with DTT when indicated. Radioactive proteins were visualized by SDS-PAGE and autoradiography. Total samples contain 10% of the material used per immunoprecipitation reaction. Arrows depict radiolabeled fractions of Mia40 in a complex with *At*Erv1 or as a monomer upon reduction with DTT, both pulled down with the HA-tagged *At*Erv1. **B**–**E** Radiolabeled Tim9 (B, C) or Tim17 (D, E) were incubated for 2 min with isolated Δ*erv1* mitochondria containing *Sc*Erv1 (B, D) or *At*Erv1 (C, E). Mitochondria were treated with 400 μM cleavable cross-linker dithiobis succinimidyl propionate for 5 min at 25 °C, incubated in NEM-containing buffer for 20 min on ice and finally lysed with 1% SDS. The extract was used for immunoprecipitation with Erv1-specific antibodies or with PIS. Disulfide bonds were reduced with DTT. Radioactive proteins were visualized by SDS-PAGE and autoradiography. Total samples contain 10% of the material used per immunoprecipitation reaction. Arrows depict radiolabeled proteins pulled down with Erv1. (EPS 23207 kb)

Additional file 5: Figure S5. Related to Fig. 5 CCMH appears not to interact with Mia40 during import into *At*Erv1 mitochondria. **A** The experiment was performed as described for Fig. 5a, with the exception that Mia40-specific antibodies were used here. Radiolabeled *A. thaliana* CCMH was incubated for 2 min with isolated mitochondria from the *At*Erv1-expressing Δ*erv1* mutant. Mitochondria were treated with 400 μM cleavable cross-linker dithiobis succinimidyl propionate for 5 min at 25 °C and lysed with 1% SDS. The extract was used for immunoprecipitation with a combination of Mia40-specific antibodies or with pre-immune serum. The crosslinker was cleaved with DTT when indicated. Radiolabeled protein was visualized by autoradiography. Total samples contain 10% of the material used per immunoprecipitation reaction. Note that no CCMH was immunoprecipitated here, in contrast to the significant amounts that were pulled down with *At*Erv1 (Fig. 5a). (EPS 2090 kb)

Additional file 6: Figure S6. Related to Fig. 5 Plant Erv1 facilitates import and oxidative folding of proteins in temperature-sensitive *mia40* mutants. **A** Wild-type cells and the temperature-sensitive *mia40-3* and *mia40-4* mutants harboring either an empty vector or an *At*Erv1-expressing plasmid were serially dropped on glucose-, galactose-, or glycerol-containing medium. Plates were incubated for 5 days at the indicated temperatures. The expression of plant Erv1 partially restored the growth defect of *mia40* strains at restrictive temperature. **B** The levels of the intermembrane space (IMS) proteins Mia40 and Cmc1 and of the matrix protein Mrpl40 were analyzed by western blotting in the indicated strains. Cells were cultured at permissive (22 °C) or at restrictive (34 °C) conditions before preparation of the protein extract. **C** Model of the Mia40 reaction cycle in the IMS of *Arabidopsis*. As described in this study, *At*Erv1 can directly interact with IMS proteins in order to oxidize them independently of Mia40. *At*Mia40, which is present in plants though non-essential, might improve the import reaction of certain substrates. (EPS 50673 kb)

## Abbreviations

ER: endoplasmic reticulum
IMS: intermembrane space
ITS: IMS-targeting signal
MISS: mitochondrial intermembrane space sorting signal
mmPEG_24_: methyl-polyethylene glycol_24_-maleimide
PDI: protein disulfide isomerase
