# Supplementary figures and images for: Erv1 of Arabidopsis thaliana can directly oxidize mitochondrial intermembrane space proteins in the absence of redox-active Mia40
Source: BMC Biol. 2017 Nov 8;15:106. doi: 10.1186/s12915-017-0445-8 (PMC5679390; doi:10.1186/s12915-017-0445-8)

**
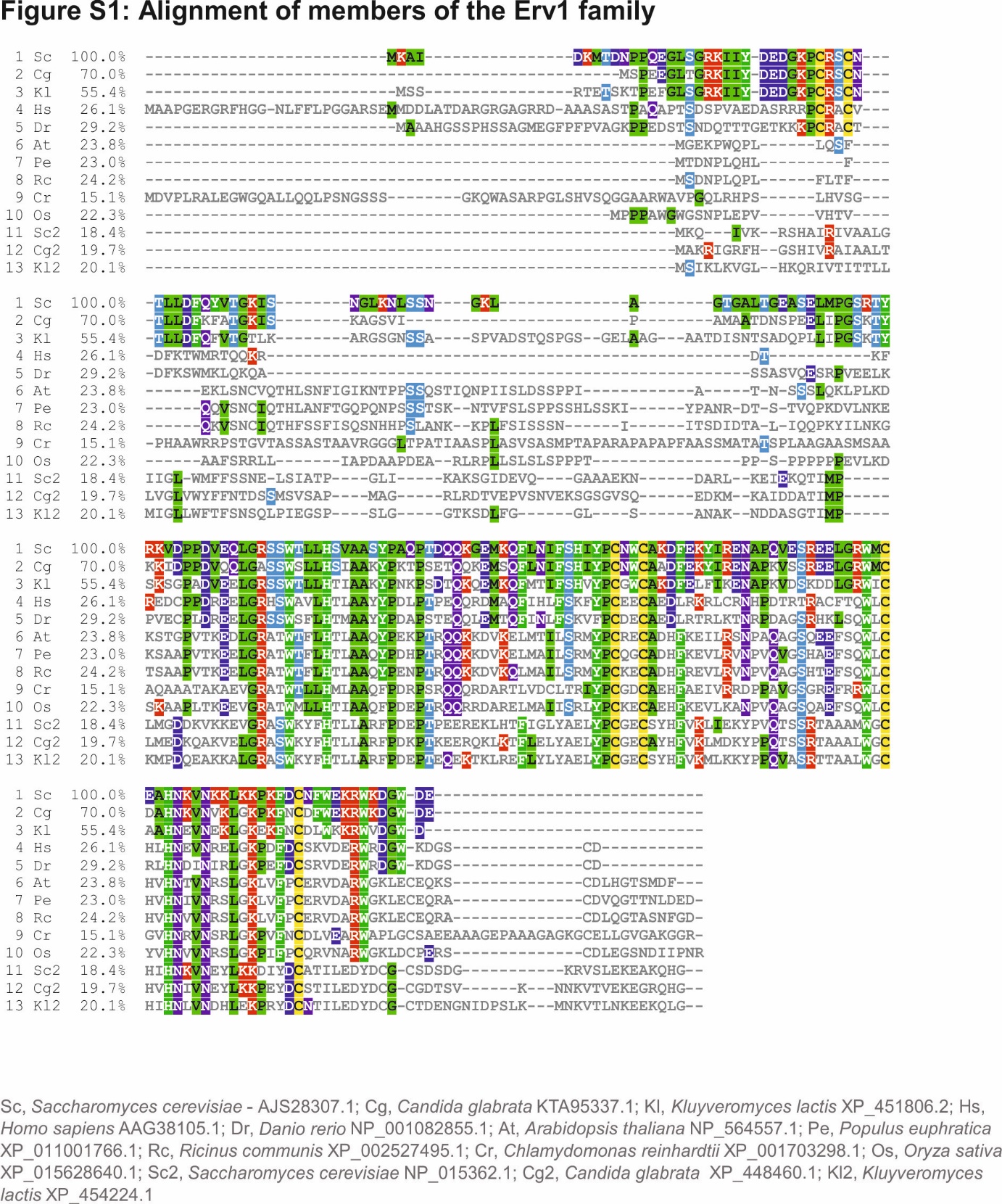
**

Supplement: Supplementary file 1 — Related to Fig. 1. Alignment of members of the Erv1 family. The sequences were compared using Clustal Omega with standard settings of the program. (DOCX 1001 kb) [file 12915_2017_445_MOESM1_ESM.docx]
